# Design and Synthesis of Acridine-Triazole and Acridine-Thiadiazole Derivatives and Their Inhibitory Effect against Cancer Cells

**DOI:** 10.3390/ijms24010064

**Published:** 2022-12-21

**Authors:** Lini Huo, Xiaochen Liu, Yogini Jaiswal, Hao Xu, Rui Chen, Rumei Lu, Yaqin Nong, Leonard Williams, Yan Liang, Zhiruo Jia

**Affiliations:** 1 College of Pharmacy, Guangxi University of Chinese Medicine, Nanning 530222, China; 2 Center for Excellence in Post-Harvest Technologies, North Carolina Agricultural and Technical State University, The North Carolina Research Campus, 500 Laureate Way, Kannapolis, NC 28081, USA; 3 College of Pharmacy, Guangxi Medical University, Nanning 530021, China

**Keywords:** acridine-triazole, acridine-thiadiazole, topoisomerase I, anti-angiogenesis, zebrafish

## Abstract

We report herein the design and synthesis of a series of novel acridine-triazole and acridine-thiadiazole derivatives. The newly synthesized compounds and the key intermediates were all evaluated for their antitumor activities against human foreskin fibroblasts (HFF), human gastric cancer cells-803 (MGC-803), hepatocellular carcinoma bel-7404 (BEL-7404), large cell lung cancer cells (NCI-H460), and bladder cancer cells (T24). Most of the compounds exhibited high levels of antitumor activity against MGC-803 and T24 but low toxicity against human normal liver cells (LO2), and their effect was even better than the commercial anticancer drugs, 5-fluorouracil (5-FU) and cis-platinum. Further, pharmacological mechanisms such as topo I, cell cycle, cell apoptosis, and neovascularization were all evaluated. Only a few compounds exhibited potent topo I inhibitory activity at 100 μM. In addition, the most active compounds with an IC_50_ value of 5.52–8.93 μM were chosen, and they could induce cell apoptosis in the G2 stage of MGC-803 or mainly arrest T24 cells in the S stage. To our delight, most of the compounds exhibited lower zebrafish cytotoxicity but could strongly inhibit the formation of zebrafish sub-intestinal veins, indicating a potential for clinical application.

## 1. Introduction

Today, cancer is one of the major health problems in the world. With the development of molecular biology and molecular pharmacology, the pathogenesis of cancer is being explored at the gene level. Pharmacological mechanisms such as signal transduction, neovascularization, telomerase, topoisomerase, cell cycle and cell apoptosis have major impacts on cancerous cells and can be used as targets in cancer therapy [1].

Acridines are an important classe of nitrogen-containing heterocyclic compounds. Due to their structural characteristics as planar tricyclic aromatic molecules, acridines intercalate tightly but reversibly to the DNA helix [2,3]. These compounds reveal a wide variety of biological activities, including anticancer [4], antimicrobial [5,6], anti-acetylcholinesterase [7], etc. A number of acridine derivatives serve as chemotherapeutic agents, especially in the field of antitumor DNA-binding agents [8]. An example of one such compound is 9-amsacrine, which has been clinically used for the treatment of leukemia [9].

Due to their beneficial characteristics, triazole and thiadiazole derivatives can serve as potential antitumor agents and thus are of pharmaceutical interest. In drug development, the triazole ring is often used to replace the amino group to reduce the resistance of some anticancer drugs and enhance their anticancer activity [10]. Thiadiazole groups are commonly introduced in the design of anticancer drugs because of their high anticancer activity. Kumar et al. recently reported the synthesis and anticancer activity of a series of benzpyrole-thiadiazole derivatives and revealed the important role of the thiadiazole ring in cytotoxicity [11].

**Figure 1:**
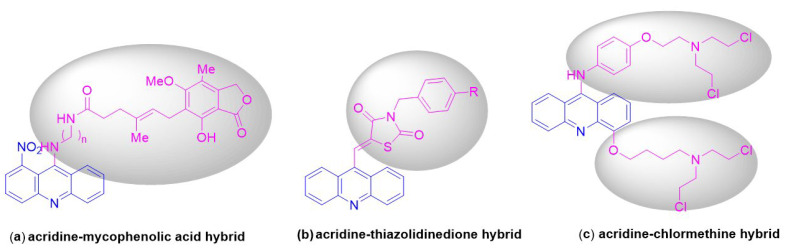
Structures of some hybrid molecules (**a**–**c**) at the 9-position of the acridine skeleton.

Designing hybrid drugs with multiple effects is a common strategy in the recent search for new anticancer drugs [12]. In recent years, many structurally diverse hybrid molecules at the 9-position of the acridine skeleton have been reported for the enhancement of anti-cancer activity. Examples of such compounds include acridine-mycophenolic acid hybrid (a) [13], acridine-thiazolidinedione hybrid [14] (b), and acridine-chlormethine hybrid (c) [15] (Figure 1).

**Figure 2:**
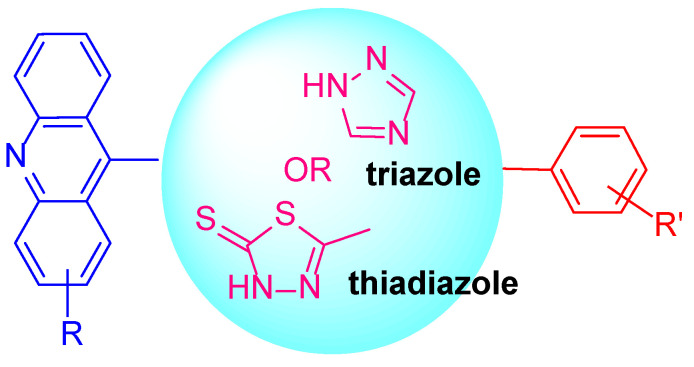
Strategy for the design of acridine-triazole or acridine-thiadiazole hybrids.

Considering these facts, our strategy was to couple an acridine and a triazole or thiadiazole nucleus to obtain a new class of compounds such as the acridine-triazole hybrid or acridine-thiadiazole hybrid (Figure 2). The anticancer activities of the synthesized compounds were assessed based on various mechanisms of action and molecular docking.

## 2. Results and Discussion

### 2.1. Chemistry

**Scheme 1:**
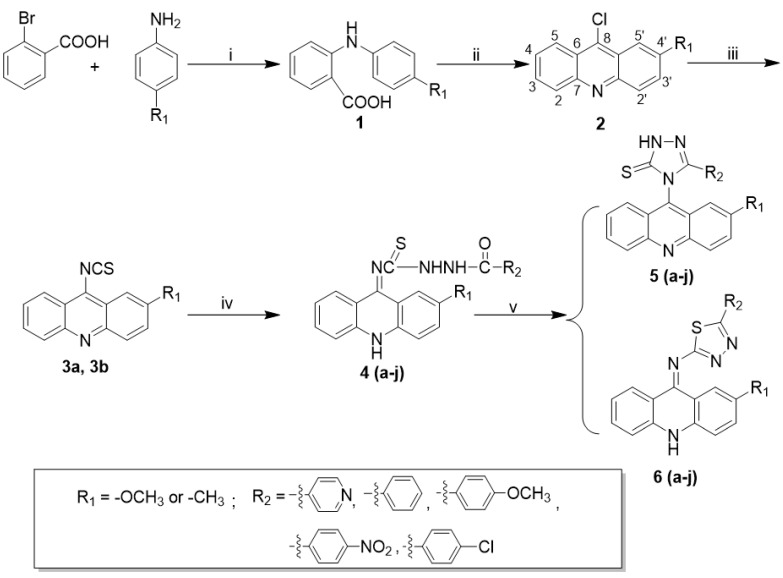
Syntheses of acridinyl derivatives. Reagents and conditions: (i) Cu, K2CO3, 140 °C; (ii) POCl3, 140 °C; (iii) NaSCN/ tetrabutylammonium bromide; (iv) 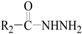; (v) Na2CO3, reflux, or 98% H2SO4, 0 °C.

The general synthetic approach for aroyl thiourea derivatives (**4**), acridinyl 1,2,4-triazole derivatives (**5**) and acridinyl 1,2,4-thiadiazole derivatives (**6**) is illustrated in Scheme 1.

The target compounds of 1,2,4-triazolethiones (**5**) and 1,2,4-thiadiazoles (**6**) were synthesized by means of a ring closure reaction using aroyl thiourea derivatives (**4**) in sodium carbonate or concentrated sulfuric acid conditions, respectively. The synthesis of aroyl thiourea derivatives (**4**) was carried out according to the known procedure of the addition of substituted hydrazides to acridin-9-yl isothiocyanate (**3**). It is important to note that the precipitate **3a** is formed at room temperature, while **3b** needs to be cooled in an ice bath. The key intermediates (**4**) were obtained in 95% EtOH without purification with a yield of 73–92% *^w^*/*_w_*.

**Figure 3:**
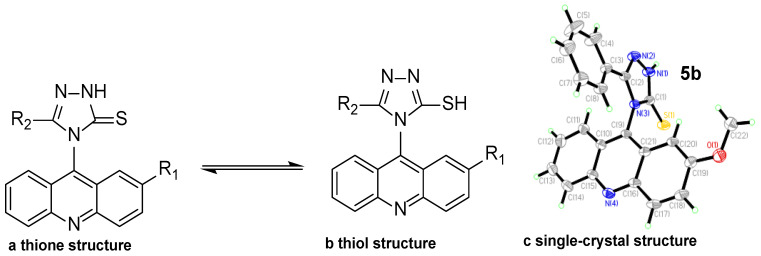
The molecular structure of compound **5b**.

As expected, auto-condensation cyclization proceeded effectively in the refluxing condition of 5% Na_2_CO_3_ or 98% concentrated sulfuric acid in an ice bath. It is reported that acridinyl 1,2,4-triazole derivatives (**5**) possibly exist in one of two tautomeric forms (Figure 3), thione (**a**) or thiol (**b**) [16]. And the thione form (**a**) was established by comparison of the HSQC and HMBC spectra and DFT calculations. To further confirm the structure of our synthesized products, a single crystal of compound **5b** was cultivated in absolute ethyl alcohol, and the molecular structure was confirmed as indicated in Figure 3c. The corresponding single crystal structural data for compound **5b** is provided in the supporting information (CCDC 2214949).

**Figure 4:**
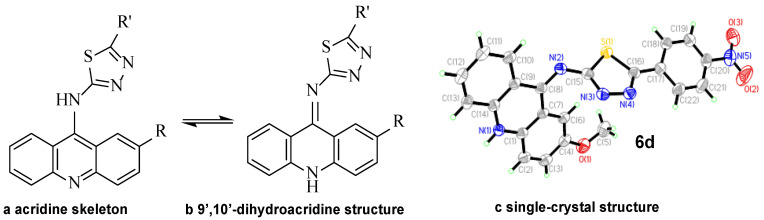
The molecular structure of compound **6d**.

**Figure 5:**
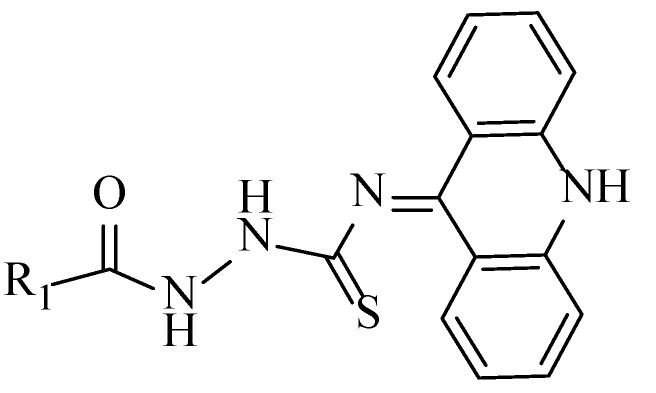
Reported structure of acridine thiosemicarbazides [13].

The success of the cyclization of compound **6** mainly depended on reaction temperature and reaction time. The reaction temperature had to be maintained below 0 °C. When R_2_ was an electron-withdrawing group such as pyridyl and nitrophenyl, the reaction time had to be extended almost to 48 h. Interestingly, the final structure of compound **6** was not the desired acridine skeleton (a, Figure 4) for the compound. The N-10 atom of the acridinyl moiety captured a proton and thus resulted in the formation of a 9′,10′-dihydroacridine structure (b, Figure 4), which was verified through X-ray crystallographic analysis (c). The corresponding single crystal structural data of compound **6d** is provided in the supporting information (CCDC 2214923). The exchangeable NH protons of acridine thiosemicarbazides are reported in the literature (Figure 5) [16].

### 2.2. In-Vitro Anticancer Activity Assay and Structure-Activity Analysis

All newly synthesized acridinyl derivatives (**4**–**6**) were screened for their anticancer activities in comparison to the reference compounds, 5-FU and cis-platinum. Compounds **4**–**6** were tested for their in vitro antitumor activities against HFF, MGC-803, BEL-7404, NCI-H460, and T24 tumor cell lines, and human normal liver cells (LO2), and the results are shown in Table 1. Most of the compounds had strong selective potency against MGC-803 and T24 cancer cells. In the MGC-803 cell line assay, almost all of the compounds displayed better cytotoxicity than the positive control 5-FU (IC_50_ = 30.45 ± 2.87 μM), with an IC_50_ of 5.52–34.99 μM. This indicates that the introduction of the triazole and thiadiazole groups on the acridine skeleton could improve the antitumor activity against MGC-803. In addition, except for compounds **4c**, **4d**, **4e**, **4i**, **4j**, **5a**, **5g**, **6b**, **6d**, and **6i**, almost all of the compounds demonstrated better cytotoxicity inhibition than cis-platinum (IC_50_ = 15.97 ± 1.53 μM). Particularly, the IC_50_ values of compounds **5d**, **5g**, **5i**, **6g**, **6e**, and **6h** were all below 10 μM, and the IC_50_ of them were 5.52 ± 1.04 μM, 8.5 ± 1.85 μM, 8.92 ± 0.99 μM, 9.01 ± 1.32 μM, 9.95 ± 1.03 μM, and 6.85 ± 0.84 μM, respectively. In the T24 cell line assay, many compounds, especially the series of compound **4**, had significant activity against T24. This implies that there is a significant increase in potency after the introduction of the aroyl thiourea group. Among these compounds, R_1_ = -CH_3_ and R_2_ = -OCH_3_ might help to improve the antitumor activity of acridine nuclear, such as compounds **4h**, **5h** and **6h**, all of which exhibited the best inhibition compared with other analogues, with IC_50_ values of 8.05 ± 1.06, 11.25 ± 1.16, and 8.93 ± 1.25 μM, respectively. In particular, compounds **4h** and **6h** had better antitumor activities than the two commercial anticancer drugs 5-FU (IC_50_ = 32.04 ± 1.23) and cis-platinum (IC_50_ = 9.13 ± 1.54 μM). To our delight, most 1,2,4-triazolethiones (**5**) and 1,2,4-thiadiazoles (**6**) have low toxicity to LO2 compared with the positive control. Compounds **5d** and **6h** were the most active but had lower toxicities than 5-FU and *cis*-platinum. Therefore, compounds **5d** and **6h** or **4h** and **6h** exhibited good cytotoxicity inhibition against MGC-803 or T24 cancer cells and were selected for further exploration to identify their mechanisms of cancer cell growth inhibition.

### 2.3. Antitumor Mechanism Studies

#### 2.3.1. Apoptosis and Cell-Cycle Analysis

Apoptosis and the cell-cycle play a central role in cancer, since their induction in cancer cells is critical to a successful therapy [17,18]. Therefore, the most active compounds, including **5d** and **6h** or **4h** and **6h** were selected to study their effect on apoptosis and cell cycle profiles in the MGC80-3 or T24 cell lines, respectively.

**Figure 6:**
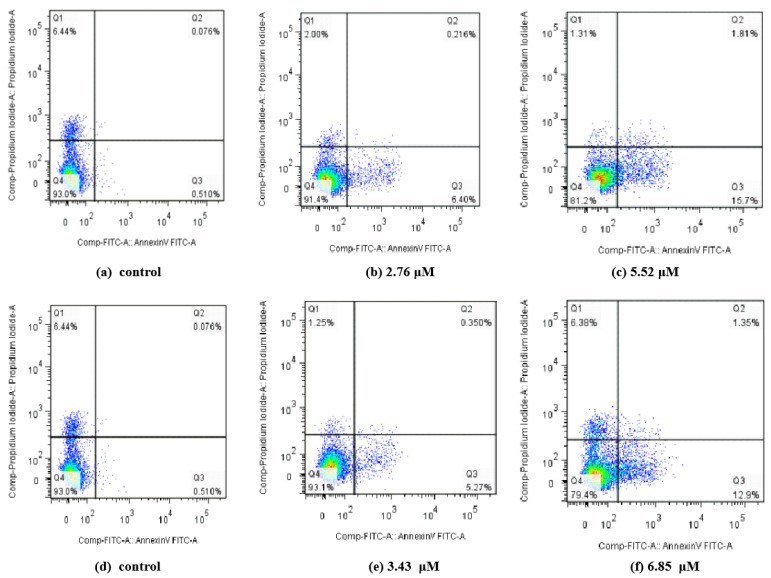
Apoptosis ratio detection of compounds **5d** and **6h** by Annexin V-FITC and PI. (**a**,**d**) The MGC80-3 cells not treated with compounds **5d** or **6h** were used as controls; (**b**,**c**) compound **5d** treated MGC80-3 cells for 24 h at concentrations of 2.76 and 5.52 μM, respectively; (**e**,**f**) compound **6h** treated MGC80-3 cells for 24 h at concentrations of 3.34 and 6.85 μM, respectively.

**Figure 7:**
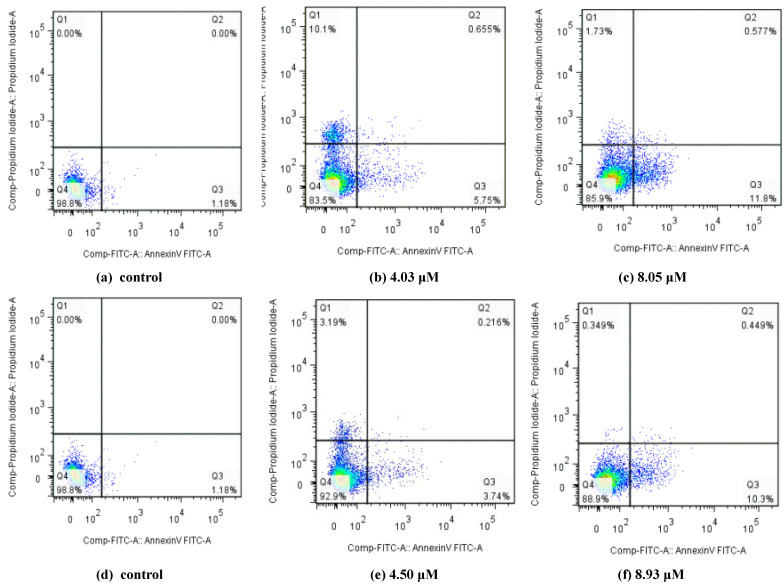
Apoptosis ratio detection of compounds **4h** and **6h** by Annexin V-FITC and PI. (**a**,**d**) The T24 cells not treated with compounds **4h** and **6h** were used as controls; (**b**,**c**) compound **4h** treated T24 cells for 24 h at concentrations of 4.03 and 8.05 μM, respectively; (**e**,**f**) compound **6h** treated T24 cells for 24 h at concentrations of 4.50 and 8.93 μM, respectively.

The apoptosis ratios of MGC80-3 or T24 cell lines induced by the selected compounds at the concentration of IC_50_ and 0.5 IC_50_ were quantitatively determined by flow cytometry. Four quadrant images (Q1, Q2, Q3 and Q4) were observed by flow cytometric analysis. The results of apoptosis ratios (including the early and late apoptosis ratios) after 12 h are presented in Figure 6 (MGC80-3) and Figure 7 (T24). Figure 6 revealed that compounds **5d** and **6h** could induce apoptosis in MGC80-3 cells in a concentration dependent manner. The apoptosis percentage of compound **5d** measured at different concentrations were found to be 6.616% (2.76 μM) and 17.51% (5.52 μM), while the value for control was 0.586%. Treatment was also accompanied by a decrease in the percentage of live cells, with values of 93.0% in control and 81.2% in treated cells. After treatment with compound **6h**, 5.62% (3.43 μM) and 14.25% (6.85 μM) of the cells were apoptotic. These were higher percentages than the one observed in the control (0.586%)**.** These results further demonstrate that apoptosis was induced by compounds **5d** and **6h** in addition to cell proliferation inhibition. From the results of Figure 7, compounds **4h** and **6h** led to an increase in the number of apoptotic cells in T24 with the increase of the concentration (from 0.5 IC_50_ to IC_50_), and their apoptosis ratios at their IC_50_ concentrations were increased to 12.377% and 10.749%, respectively, when compared with the control (1.18%). All compounds had little effect on late apoptosis of MGC80-3 or T24, and some normal cells were found to be necrotic in Q1 region. The results evidently illustrate that representative compounds **5d** and **6h** or **4h** and **6h** could suppress cell proliferation by inducing apoptosis in the early apoptotic period.

**Figure 8:**
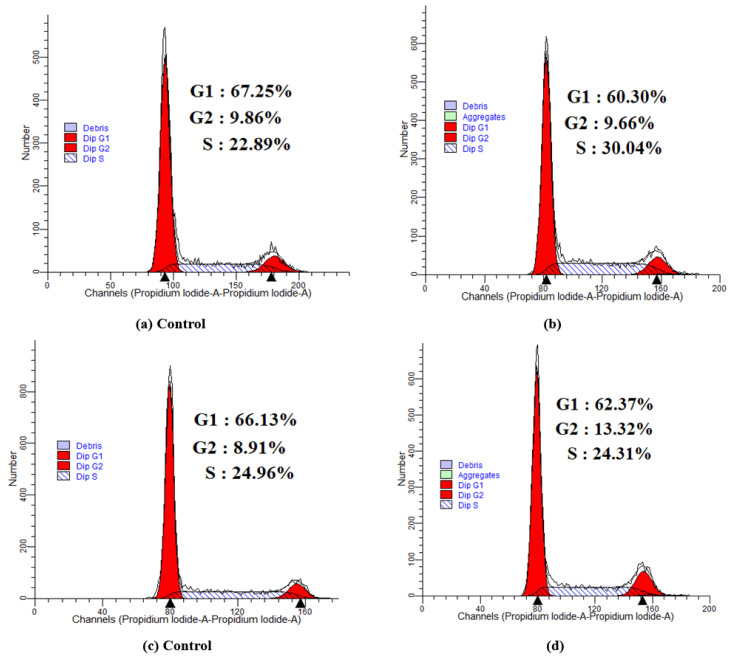
Cell cycle analysis of compound **4h** treated T24 cells (**b**) and compound **5d** treated MGC80-3 cells (**d**) at their IC_50_ concentrations (8.05 μM and 5.52 μM) for 48 h. And the T24 and MGC80-3 cells not treated with compounds **4h** and **5d** were used as control, (**a**,**c**). (G1: Prophase of DNA synthesis; S: stage of dna synthesis; G2: Late stages of DNA synthesis).

The cell cycle distributions of T24 and MGC80-3cells after 48 h of treatment with the most active compounds, **4h** and **5d,** at their IC_50_ concentrations are shown in Figure 8. Compared to control, both compounds **4h** and **5d** interfered with the cell cycles of T24 and MGC80-3 cells, respectively. As shown in Figure 8a,b, the S-phase population of T24 cells increased by 30.04% compared to the control cells (22.89%), indicating that compound **4h** might inhibit the growth of tumor cells by arresting the cells in S phase during the DNA synthesis period. However, compound **5d** could induce a significant cell cycle arrest in the G2 phase, resulting in a concomitant population increase (13.32%) compared with the control cells (8.91%) at a concentration of 5.52 μM (Figure 8c,d). These results suggest that compound **5d** may inhibit the growth of tumor cells by arresting cells in the G2 phase in the late stage of DNA synthesis.

#### 2.3.2. Evaluation of Topo I Inhibitory Activity

**Figure 9:**
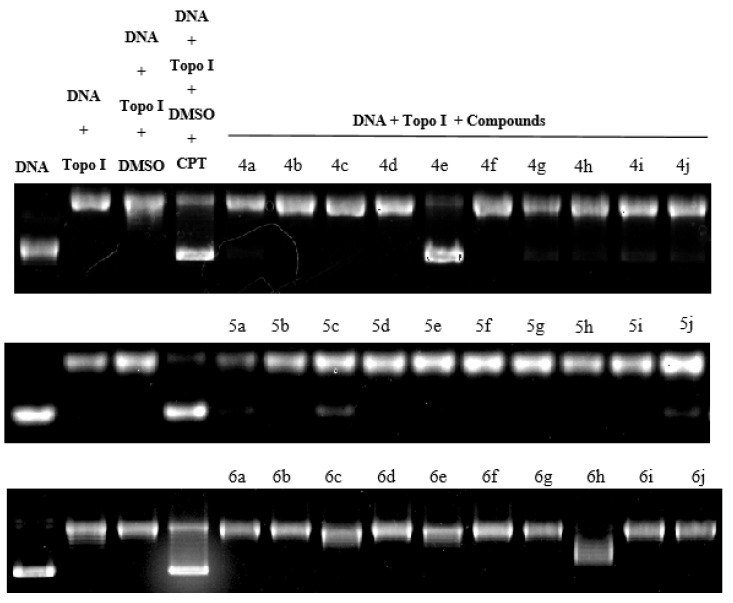
DNA topoisomerase I (Topo I) inhibitory activity of CPT and all target compounds (**4a**–**4j, 5a**–**5j, 6a**–**6j**) at 1 mM.

**Figure 10:**
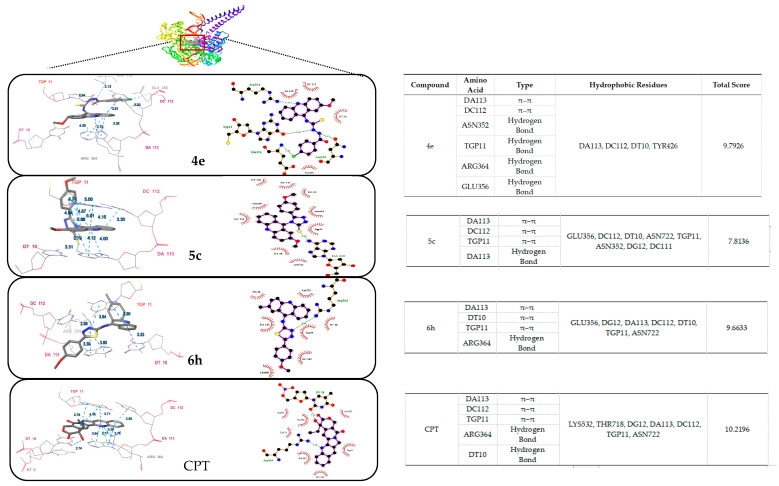
The best pose of the binding mode of compounds (**4e**, **5c**, **6h** and **CPT**) with DNA Topo I complex (PDB:1T8I).

DNA topoisomerase I (topo I) has become the main molecular target in anticancer drugs on account of its significance in all living organisms, participating in replication, transcription, recombination, and repair in many cellular metabolic processes. The topo I inhibitory activity of the compounds with the known topo I inhibitor camptothecin (CPT) is depicted in Figure 9. Only compounds **4e**, **5c**, and **6h** exhibit potent topo I inhibitory activity at 100 µM. Compounds that have little to no inhibitory activity may have other mechanisms for their anticancer effects. Molecular docking studies of the selected compounds were carried out by the Surflex-Dock algorithm of Sybyl-X 2.0 (Tripos Inc., St. Louis, MI, USA). The molecular docking approach was verified by our previously published methods (RMSD (root-mean-square deviation) value was 0.4438 Å) [19]. The binding affinities of protein-ligand complexes were expressed as a total score and shown in Figure 10. compounds **4e**, **5c** and **6h** exhibited good binding affinities, with total scores of 9.79, 7.81 and 9.66, respectively. Potent Topo I inhibitory activity of these compounds may be attributed to the formation of hydrophobic residue, hydrogen bond, and π–π stacking with the same amino acid residue DA113, DC112, TGP11 as CPT.

#### 2.3.3. Toxicity and Anti-angiogenesis in the Zebrafish Model

**Figure 11:**
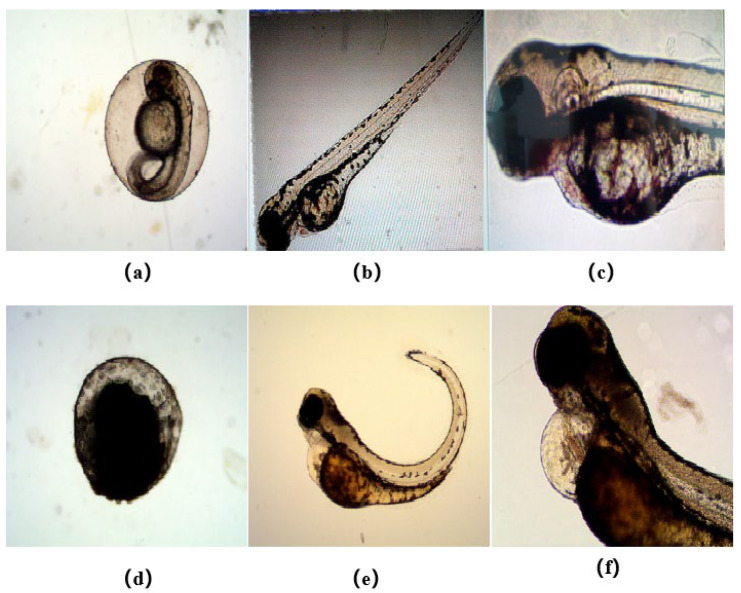
Normal and abnormal zebrafish embryos (**a**) Normal zebrafish embryos (**b**) Normal zebrafish (**c**) Normal pericardium of zebrafish (**d**) Embryo necrosis (**e**) Severe angulation of the spine (**f**) Severe pericardial edema.

Many antitumor drugs inevitably have side effects on normal cells, such as bone marrow suppression, liver and kidney injury, and abnormal blood cells. Therefore, in order to improve the possibility of clinical application of acridine-heterocyclic derivatives, the effective and low-toxicity antitumor drugs were screened using a zebrafish model. In this experiment, 2% DMSO was used to dissolve the target products, and the abnormal rate (MAR) and mortality rate (MOR) of zebrafish embryos (72 hpf) were used for statistics. At different concentrations (1–2 mg/mL) of the selected compounds, various deformities were observed, such as failure to hatch, embryo necrosis, severe angulation of the spine and severe pericardial edema (Figure 11). The mortality and malformation rates of embryos increased with a dose-effect relationship (Table 2). Almost all compounds in the compound **4** series were toxic. Particularly, compounds **4a**, **4b**, **4f** and **4h** had a total mortality and malformation rate of 100% at 2.0 mg.L^−1^, exhibiting the strongest embryonic toxicity. It is worth mentioning that compounds **5d** and **5h** displayed high levels of antitumor activities but were less toxic to zebrafish embryos. At the highest concentration of 2.0 mg.L^−1^, the mortality rate of zebrafish embryos was close to 0% and the malformation rate was less than 15%. Moreover, there was hardly any toxicity observed in compound **6** at lower concentrations (1.0 mg.L^−1^). Compound **6i** exhibited very low toxicity at a high concentration of 2.0 mg.L^−1^ with 0% mortality rate and 25% malformation rate.

**Figure 12:**
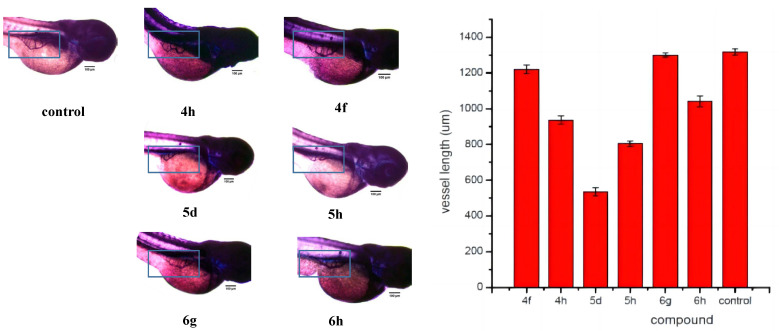
Effects of representative compounds (**4f, 4h, 5d, 5h**, **6g** and **6h**) and control on the subintestinal veins (SIVs) length of 72 hpf zebrafish embryos (x ± s, *n* = 12), *p* < 0.05.

Currently, the zebrafish has emerged as a valuable model organism to substitute traditional models for studying angiogenesis inhibitors [20]. The genes of zebrafish show 70–80% similarity to humans, and the vascular structure of zebrafish has high similarity to that of other vertebrates [21,22,23]. Therefore, the subintestinal veins (SIVs) in the zebrafish embryos are used as evaluation indicators for anti-angiogenesis inhibitors. In this study, NBT/BCIP vascular staining was used to observe the angiogenesis effect of representative drugs (**4h**, **4f**, **5d**, **5h**, **6g** and **6h**) in a zebrafish model. As shown in Figure 12**,** SIVs grew well in the blank group, naturally extending into a network in the abdomen with many branches. The length of SIVs of zebrafish was measured by Image J software and is shown in Figure 10b. Compared with the blank control group, the area of the meshed pattern vessel and the number of vascular branches in the network decreased after the administration of compounds **4h**, **5d**, **5h** and **6h.** Among these compounds, compounds **5d** and **5h** exhibited the strongest antiangiogenic effects that led to a nearly 50% reduction in the vessel length compared to the mean vessel length for the controls. Compounds **4a** and **6g** could reduce the area of blood vessels, but at the same time, additional blood vessels were formed on the blood vessel edge.

## 3. Materials and Methods

All commercially available chemicals were reagent grade and bought from Aladdin Reagent Co., LTD (Shanghai, China); NBT/BCIP kit was bought from Tiangen Biochemical Technology Co., LTD (Shanghai, China); AnnexinV-FITC apoptosis detection kit was bought from Nanjing KGI Biotechnology Development Co., Ltd. (Nanjing, China); The spectra such as NMR, MS, and IR were all evaluated and recorded on a Bruker DRX-400 (^1^H: 400 MHz, ^13^C: 100 MHz) (Rheinstetten, Germany), a Thermo Fisher LCQ Fleet (ESI) instrument (Waltham, MA, USA), and FT-IR Thermo Nicolet Avatar 360 using a KBr pellet (Waltham, MA, USA). And the melting points were measured by the XT-4 A melting point apparatus (Shanghai, China) without correction. Other instruments include BD FACSAria II Flow cytometer (Franklin Lakes, NJ, USA), MCO96 carbon dioxide incubator (Osaka, Japan) and Bio Tek EL × 800 microplate reader (Winooski, VT, USA), etc.

### 3.1. Synthesis Methods

#### 3.1.1. Synthesis of N-phenyl-o-aminobenzoic acid (**1**) and 9-chlorine acridine (**2**)

The synthesis of N-phenyl-o-aminobenzoic acid (**1**) and 9-chlorine acridine (**2**) was carried out according to our previously published procedure, with slight modifications [24]. Compound **1** could proceed to the next step without further purification.

2-methoxyl-9-chlorine acridine (**2a**): Yellow-green needle crystal, yield 85.2%, m.p. 158–159 °C. ESI-MS m/z: 244 ([M + H]^+^); ^1^H NMR (CDCl_3_, 400 MHz) 8.00 (dd, 2H, *J* = 8.00, ArH), 7.93 (d, 2H, *J* = 8.20, ArH), 7.55–7.60 (m, 2H, ArH), 7.50 (d, 1H, *J* = 8.40, ArH), 7.30 (s, 1H, ArH), and 3.73 (s, 3H, -OCH_3_).

2-methyl-9-chlorine acridine (**2b**): Pale green needle crystal, yield 78.5%, m.p. 122–123 °C. ESI-MS m/z: 228 ([M + H])^+ 1^H NMR (CDCl_3_, 400 MHz), δ: 8.05 (dd, 2H, *J* = 8.00, ArH), 8.00 (d, 2H, *J* = 9.20, ArH), 7.61–7.68 (m, 2H, ArH), 7.50 (d, 1H, *J* = 5.40, ArH), 7.43 (s, 1H, ArH), 2.35 (s, 3H, -CH_3_).

#### 3.1.2. Synthesis of 9-acridinyl Isothiocyanate (**3**)

To a solution of chlorine acridine **2** (5 mmol) in acetone (50 mL), NaSCN (0.81 g, 10 mmol) and tetrabutylammonium bromide (0.32 g, 1 mmol) were added, and the mixture was then refluxed at 60 °C for 1 h. After cooling to room temperature, crystals of **3a** were immediately precipitated in the reaction mixture, and crystals of **3b** were precipitated in an ice bath. At the end of the procedure, the crystals were filtered, washed with water, and dried under vacuum, and no further purification was carried out.

2-methoxyl-9-acridinyl isothiocyanate (**3a**): bright yellow crystal, yield 88.0%, m.p. 149–150 °C; ESI-MS m/z: 267 ([M + H]^+^); ^1^H NMR (CDCl_3_, 400 MHz), δ: 8.25 (d, 2H, *J* = 8.50, ArH), 8.15 (d, 1H, *J* = 9.20, ArH), 7.77–7.81 (q, 1H, ArH), 7.66–7.68 (t, 1H, ArH), 7.51 (d, 1H, *J* = 8.00, ArH), 7.40 (s, 1H, ArH), 4.08(s, 3H, -OCH_3_); ^13^C NMR (CDCl_3_, 100 MHz) δ: 158.66, 130.48, 127.54, 127.04, 123.48, 122.62, 122.28, 98.50, 55.90; IR (KBr) ν: 2967, 2098 (-N=C=S), 1356–1557 cm^−1^.

2-methyl-9-acridinyl isothiocyanate (**3b**): faint yellow needle crystal, yield 94%, m.p. 128–129 °C; ESI-MS m/z: 351 ([M + H]^+^); ^1^H NMR (CDCl_3_, 400 MHz), δ: 8.26–8.28 (m, 2H, ArH), 8.15 (d, 1H, *J* = 8.40 Hz, ArH), 8.04 (s, 1H, ArH), 7.83 (t, 1H, ArH), 7.64–7.70 (m, 2H, ArH), 7.40 (s, 1H, ArH), 2.67 (s, 3H, -CH_3_); ^13^C NMR (CDCl_3_, 100 M Hz) δ: 137.59, 130.46, 127.08, 125.21, 122.92, 122.21, 121.17, 22.12; IR (KBr) ν: 2903, 2143 (-N=C=S), 1411–1630 cm^−1^.

#### 3.1.3. General Procedure for the Synthesis of Acridinyl Aroyl Thiourea Derivatives **4a**–**4f**

To a solution of 9-isothiocyanatoacridine **3** (2mmol) in absolute ethyl alcohol (60 mL), the appropriate substituted hydrazides (2 mmol) were added, and the reaction mixture was refluxed until the reactants had been consumed (monitored by TLC). The precipitate of **4a**–**4f** was prepared, filtered off, washed with 95% ethyl alcohol, and dried at room temperature.

1-2′-methoxyl acridinyl-3-4′-pyridinamide thiourea (**4a**): Yellow powder, Yield 91%, m.p. 200–206 °C; ESI-MS m/z: 404 ([M + H]^+^); ^1^H NMR (400 MHz, DMSO-*d^6^*), δ: 11.26 (br, s, 1H, -NH), 10.41 (br, s, 1H, -NH), 10.22 (br, s, 1H, -NH), 8.80 (s, 1H, ArH), 8.05–8.15 (m, 2H, ArH), 7.93 (s, 1H, ArH), 7.77 (t, 1H, ArH), 7.63 (t, 1H, ArH), 8.15 (d, 1H, *J* = 8.40 Hz, ArH), 8.04 (s, 1H, ArH), 7.83 (t, 1H, ArH), 7.53 (d, 1H, *J* = 9.20 Hz, ArH), 7.45 (s, 1H, ArH), 4.02 (s, 3H, -OCH_3_); ^13^C NMR (DMSO-*d^6^*, 100 MHz) δ: 183.19, 167.30, 165.41, 158.40, 150.74, 148.72, 141.00, 139.61, 131.03, 129.59, 125.82, 122.40, 113.46, 109.76, 56.09; IR (KBr) ν: 3108, 2948 (N–H), 1695 (-C=O), 1291 (-C=S) cm^−1^.

1-2′-methoxyl acridinyl-3-benzoyl thiosemicarbazides (**4b**): Yellow powder, Yield 76%, m.p. 190–192 °C; ESI-MS m/z: 403 ([M + H]^+^); ^1^H NMR (400 MHz, DMSO-*d^6^*), δ: 10.96 (br, s, 1H, -NH), 10.40 (br, s, 1H, -NH), 10.10 (br, s, 1H, -NH), 8.01–8.14 (m, 4H, ArH), 7.76 (s, 2H, ArH), 7.52–7.58 (m, 6H, ArH), 4.02 (s, 3H, -OCH_3_); ^13^C NMR (101 MHz, DMSO-*d^6^*) δ: 183.14, 166.84, 164.80, 157.06, 156.21, 144.80, 141.24, 139.31, 132.72, 129.80, 128.80, 127.28, 125.95, 122.46, 122.11, 112.10, 111.51, 56.22; IR (KBr) ν: 3102, 2941 (-N—H), 1686 (-C=O), 1289 (-C=S) cm^−1^.

1-2′-methoxyl acridinyl-3-4′-methoxy benzoyl thiosemicarbazides (**4c**): Yellow powder, Yield 87%, m.p. 202–203 °C; ESI-MS m/z: 455 ([M + Na]^+^); ^1^H NMR (400 MHz, DMSO-*d^6^*), δ: 10.81 (br, s, 1H, -NH), 10.37 (br, s, 1H, -NH), 10.04 (br, s, 1H, -NH), 8.02–8.09 (m, 4H, ArH), 7.32–7.87 (m, 6H, ArH), 7.06 (s, 1H, ArH), 3.82 (s, 3H, -OCH_3_), 3.32 (s, 3H, -OCH_3_); ^13^C NMR (101 MHz, DMSO-*d^6^*) δ: 182.97, 166.60, 162.03, 156.30, 153.82, 140.77, 136.15, 132.16, 129.58, 128.77, 127.25, 125.82, 121.52, 111.00, 108.32, 55.90; IR (KBr) ν: 3107, 2945 (-N—H), 1677 (-C=O), 1256 (-C=S) cm^−1^.

1-2’-methoxyl acridinyl-3-4′-nitro benzoyl thiosemicarbazides (**4d**): Yellow powder, Yield 94%, m.p. 223–227 °C; ESI-MS m/z: 448 ([M + H]^+^); ^1^H NMR (400 MHz, DMSO-*d^6^*), δ: 11.30 (br, s, 1H, -NH), 10.45 (br, s, 1H, -NH), 10.22 (br, s, 1H, -NH), 8.38 (s, 2H, ArH), 8.26 (s, 2H, ArH), 8.02–8.16 (m, 2H, ArH), 7.53-7.63 (m, 4H, ArH), 7.46 (s, 1H, ArH), 4.02 (s, 3H, -OCH_3_); ^13^C NMR (101 MHz, DMSO-*d^6^*) δ: 183.22, 167.59, 162.87, 157.42, 149.87, 147.71, 146.73, 140.26, 138.66, 131.49, 131.11, 129.64, 125.67, 124.67, 124.15, 123.98, 117.74, 110.08, 100.87, 56.20; IR (KBr) ν: 3105, 2947 (-N—H), 1697 (-C=O), 1527 (-C=S) cm^−1^.

1-2’-methoxyl acridinyl-3-4’-nitro benzoyl thiosemicarbazides (**4d**): Yellow powder, Yield 94%, m.p. 223–227 °C; ESI-MS m/z: 448 ([M+H]^+^); ^1^H NMR (400 MHz, DMSO-*d^6^*), δ: 11.30 (br, s, 1H, -NH), 10.45 (br, s, 1H, -NH), 10.22 (br, s, 1H, -NH), 8.38 (s, 2H, ArH), 8.26 (s, 2H, ArH), 8.02-8.16 (m, 2H, ArH), 7.53–7.63 (m, 4H, ArH), 7.46 (s, 1H, ArH), 4.02 (s, 3H, -OCH_3_); ^13^C NMR (101 MHz, DMSO-*d^6^*) δ: 183.22, 167.59, 162.87, 157.42, 149.87, 147.71, 146.73, 140.26, 138.66, 131.49, 131.11, 129.64, 125.67, 124.67, 124.15, 123.98, 117.74, 110.08, 100.87, 56.20; IR (KBr) ν: 3105, 2947 (-N—H), 1697 (-C=O), 1527 (-C=S) cm^−1^.

1-2′-methyl acridinyl-3-4′-pyridinamide thiourea (**4f**): Orange powder, Yield 82%, m.p. 176–180 °C; ESI-MS m/z: 410 ([M + Na]^+^); ^1^H NMR (400 MHz, DMSO-*d^6^*), δ: 11.59 (br, s, 1H, -NH), 10.99 (br, s, 1H, -NH), 10.21 (br, s, 1H, -NH), 8.81 (s, 2H, ArH), 7.89 (s, 3H, ArH), 7.42–7.89 (m, 6H, ArH), 2.33 (s, 3H, -CH_3_); ^13^C NMR (101 MHz, DMSO-*d^6^*) δ: 183.47, 167.14, 155.61, 150.76, 140.45, 140.16, 138.18, 133.65, 131.24, 126.31, 124.88, 117.52, 111.80, 21.93; IR (KBr) ν: 3104, 2918 (-N—H), 1556 (-C=O), 1471 (-C=S) cm^−1^.

1-2′-methyl acridinyl-3-benzoyl thiosemicarbazides (**4g**): Orange powder, Yield 82%, m.p. 171–173 °C; ESI-MS m/z: 409 [M + Na]^+^; ^1^H NMR (400 MHz, DMSO-*d^6^*), δ: 10.68 (br, s, 1H, -NH), 10.41 (br, s, 1H, -NH), 10.13 (br, s, 1H, -NH), 8.58–8.75 (m, 4H, ArH), 7.42–7.57 (m, 5H, ArH), 7.39 (d, *J* = 8.5 Hz, 2H, ArH), 7.09 (s, 1H, ArH), 2.42 (s, 3H, -CH_3_); ^13^C NMR (101 MHz, DMSO-*d^6^*,) δ: 181.29, 166.81, 153.65, 150.24, 148.96, 140.25, 138.24, 133.58, 130.84, 130.35, 126.22, 125.07, 122.13, 121.38, 117.44, 116.40, 111.44, 21.25; IR (KBr) ν: 3102, 2917 (-N—H), 1569 (-C=O), 1471 (-C=S) cm^−1^.

1-2′-methyl acridinyl-3-4’-methoxy benzoyl thiosemicarbazides (**4h**): Orange-yellow powder, Yield 93%, m.p. 210–212 °C; ESI-MS m/z: 439 ([M + Na]^+^); ^1^H NMR (400 MHz, DMSO-*d^6^*), δ: 10.53 (br, s, 1H, -NH), 10.38 (br, s, 1H, -NH), 10.08 (br, s, 1H, -NH), 7.97–8.18 (m, 4H, ArH), 7.34–7.55 (m, 3H, ArH), 7.34–7.55 (m, 3H, ArH), 7.07 (s, 1H, ArH), 3.44 (s, 3H, -OCH_3_), 2.42 (s, 3H, -CH_3_); ^13^C NMR (101 MHz, DMSO-*d^6^*) δ: 181.09, 166.30, 153.39, 148.81, 140.15, 138.18, 130.79, 130.04, 126.17, 125.32, 121.21, 117.39, 114.12, 111.37, 55.89, 22.24; IR (KBr) ν: 3094, 2914 (-N—H), 1556 (-C=O), 1471 (-C=S) cm^−1^.

1-2′-methyl acridinyl-3-4’-nitro benzoyl thiosemicarbazides (**4i**): Orange-yellow powder, Yield 73%, m.p. 187–188 °C; ESI-MS m/z: 457 ([M + Na]^+^); ^1^H NMR (400 MHz, DMSO-*d^6^*), δ: 11.56 (br, s, 1H, -NH), 10.76 (br, s, 1H, -NH), 10.15 (br, s, 1H, -NH), 8.41 (d, *J* = 8.5 Hz, 2H), 7.96–8.26 (m, 4H, ArH), 7.26–7.57 (m, 4H, ArH), 7.11 (s, 1H, ArH), 2.44 (s, 3H, -CH_3_); ^13^C NMR (101 MHz, DMSO-*d^6^*) δ: 183.42, 166.78, 155.67, 149.20, 140.24, 140.14, 131.87, 130.89, 130.41, 124.68, 117.93, 111.56, 22.50; IR (KBr) ν: 3095, 2918 (-N—H), 1598 (-C=O), 1483 (-C=S) cm^−1^.

1-2′-methyl acridinyl-3-4′-chloro benzoyl thiosemicarbazides (**4j**): Orange-yellow powder, Yield 86%, m.p. 178–179 °C; ESI-MS m/z: 443 ([M + Na]^+^); ^1^H NMR (400 MHz, DMSO-*d^6^*), δ: 10.77 (br, s, 1H, -NH), 10.48 (br, s, 1H, -NH), 10.08 (br, s, 1H, -NH), 8.18–8.34 (m, 4H, ArH), 7.57–7.83 (m, 3H, ArH), 7.33–7.57 (m, 3H, ArH), 7.07 (s, 1H, ArH), 2.42 (s, 3H, -CH_3_); ^13^C NMR (101 MHz, DMSO-*d^6^*) δ: 182.61, 166.39, 155.38, 149.09, 140.26, 139.92, 131.03, 130.08, 129.29, 124.20, 117.53, 116.31, 111.58, 104.34, 22.83; IR (KBr) ν: 3094, 2915 (-N—H), 1567 (-C=O), 1480 (-C=S) cm^−1^.

#### 3.1.4. General Procedure for the Synthesis of Acridinyl 1,2,4-triazole Derivatives **5a**–**5f**

The appropriate acyl thiosemicarbazides (**4a**–**4i**, 1 mmol) and 5% aqueous sodium carbonate (40 mL) were refluxed for 5 h. After cooling, the precipitate was filtered off and the filtrate was acidified by hydrochloric acid to a pH of 2. The precipitates were formed, filtered off and then crystallized from ethyl alcohol.

4-(2-methoxyacridin-9-yl)-5-(pyridin-4-yl)-2,4-dihydro-3H-1,2,4-triazole-3-thione (**5a**): Light yellow powder, Yield 75%, m.p. 272–273 °C; ESI-MS m/z: 384 ([M + H]^+^); ^1^H NMR (400 MHz, DMSO-*d^6^*), δ: 14.79 (br, s, 1H, -NH), 8.38 (dd, *J* = 4.6, 1.5 Hz, 2H, ArH), 8.33–8.11 (m, 2H, ArH), 7.94–7.76 (m, 1H, ArH), 7.08 (dd, *J* = 4.6, 1.6 Hz, 2H, ArH), 6.87 (d, *J* = 2.6 Hz, 1H), 3.86 (s, 3H, -OCH_3_); ^13^C NMR (100 MHz, DMSO-*d^6^*) δ: 169.99, 159.21, 157.94, 150.89, 149.46, 134.90, 130.31, 129.15, 126.69, 125.10, 124.89, 120.97, 56.52; IR (KBr) ν: 3069, 2906, 2745 (-N—H), 1505–1633 (-C=N),1480 (-C=S) cm^−1^.

4-(2-methoxyacridin-9-yl)-5-phenyl-2,4-dihydro-3H-1,2,4-triazole-3-thione (**5b**): Light yellow powder, 60%, m.p. 259–261 °C; ESI-MS m/z: 385 ([M + H]^+^); ^1^H NMR (400 MHz, DMSO-*d^6^*), δ: 14.56 (s, 1H, -NH), 8.22 (dd, *J* = 12.2, 9.1 Hz, 2H, ArH), 7.89–7.75 (m, 1H, ArH), 7.70–7.58 (m, 2H, ArH), 7.56 (d, *J* = 8.6 Hz, 1H, ArH), 7.26 (t, *J* = 6.6 Hz, 1H, ArH), 7.21–7.07 (m, 4H, ArH), 6.82 (d, *J* = 2.6 Hz, 1H, ArH), 3.85 (s, 3H, -OCH_3_); ^13^C NMR (100 MHz, DMSO-*d^6^*) δ: 169.50, 159.01, 151.69, 147.45, 146.68, 132.13, 131.27, 130.24, 130.13, 129.26, 128.94, 127.42, 126.58, 125.76, 124.95, 123.68, 122.58, 98.59, 56.44; IR (KBr) ν: 3056, 2912, 2749 (-N—H), 1500–1632 (-C=N), 1476 (-C=S) cm^−1^.

4-(2-methoxyacridin-9-yl)-5-(4-methoxyphenyl)-2,4-dihydro-3H-1,2,4-triazole-3-thione (**5c**): Light yellow powder, Yield 58%, m.p. 262–267 °C; ESI-MS m/z: 415 ([M + H]^+^); ^1^H NMR (400 MHz, DMSO-*d^6^*), δ: 14.45 (s, 1H, -NH), 8.23 (dd, *J* = 11.8, 9.1 Hz, 2H, ArH), 7.93–7.73 (m, 1H, ArH), 7.64 (s, 2H, ArH), 7.54 (s, 1H, ArH), 7.08 (d, *J* = 8.9 Hz, 2H, ArH), 6.80 (s, 1H, ArH), 6.70 (d, *J* = 8.9 Hz, 2H, ArH), 3.85 (s, 3H, -OCH_3_), 3.58 (s, 3H, -OCH_3_); ^13^C NMR (100 MHz, DMSO-*d^6^*) δ: 169.30, 161.33, 159.01, 151.58, 147.51, 146.73, 134.08, 132.17, 130.20, 128.95, 126.56, 125.02, 123.76, 122.59, 117.89, 114.76, 98.57, 56.41, 55.60; IR (KBr) ν: 3066, 2883, 2726 (N—H), 1500–1632 (C=N), 1476 (C=S) cm^−1^.

4-(2-methoxyacridin-9-yl)-5-(4-nitrophenyl)-2,4-dihydro-3H-1,2,4-triazole-3-thione (**5d**): Light yellow powder, Yield 40%, m.p. 253–255 °C; ESI-MS m/z: 430 ([M + H]^+^); ^1^H NMR (400 MHz, DMSO-*d^6^*), δ: 14.79 (s, 1H, -NH), 8.24 (t, *J* = 9.3 Hz, 2H, ArH), 8.02 (d, *J* = 8.9 Hz, 2H, ArH), 7.93–7.75 (m, 1H, ArH), 7.63 (dd, *J* = 9.5, 2.7 Hz, 2H, ArH), 7.58 (s, 1H, ArH), 7.44 (d, *J* = 8.9 Hz, 2H, ArH), 6.91 (s, 1H, ArH), 3.87 (s, 3H, -OCH_3_); ^13^C NMR (100 MHz, DMSO-*d^6^*) δ: 170.00, 159.22, 149.93, 148.90, 147.47, 146.78, 133.20, 132.22, 131.44, 130.25, 129.15, 128.69, 126.70, 124.93, 124.61, 123.51, 122.38, 98.64, 56.54; IR (KBr) ν: 3066, 2883, 2726 (-N—H), 1421–1633 (-C=N), 1345 (-C=S) cm^−1^.

4-(4-chlorophenyl)-4-(2-methoxyacridin-9-yl)-2,4-dihydro-3H-1,2,4-triazole-3-thione (**5e**): Light yellow powder, Yield 78%, m.p. 286–287 °C; ESI-MS m/z: 419 ([M + H]^+^); ^1^H NMR (400 MHz, DMSO-*d^6^*), δ: 14.61 (s, 1H, -NH), 8.23 (dd, *J* = 11.4, 9.2 Hz, 2H, ArH), 7.93–7.76 (m, 1H, ArH), 7.73–7.60 (m, 2H, ArH), 7.55 (d, *J* = 8.6 Hz, 1H, ArH), 7.25 (d, *J* = 8.6 Hz, 2H, ArH), 7.17 (d, *J* = 8.6 Hz, 2H, ArH), 6.85 (s, 1H, ArH), 3.86 (s, 3H, -OCH_3_); ^13^C NMR (100 MHz, DMSO-*d^6^*) δ: 169.59, 159.10, 150.72, 147.44, 146.71, 136.12, 133.51, 132.17, 130.22, 129.54, 129.11, 126.64, 124.93, 124.62, 123.57, 122.49, 98.61, 56.49; IR (KBr) ν: 3056, 2909, 2748 (-N—H), 1344–1632 (-C=N), 1503 (-C=S) cm^−1^.

4-(2-methylacridin-9-yl)-5-(pyridin-4-yl)-2,4-dihydro-3H-1,2,4-triazole-3-thione (**5f**): Light yellow powder, Yield 48%, m.p. 264–268 °C; ESI-MS m/z: 370 ([M + H]^+^); ^1^H NMR (400 MHz, DMSO-*d^6^*) δ: 14.84 (s, 1H, -NH), 8.37 (d, *J* = 6.0 Hz, 2H, ArH), 8.29 (d, *J* = 8.7 Hz, 1H, ArH), 8.23 (d, *J* = 8.9 Hz, 1H, ArH), 7.96–7.84 (m, 1H, ArH), 7.84–7.74 (m, 1H, ArH), 7.72–7.59 (m, 2H, ArH), 7.50 (s, 1H, ArH), 7.04 (d, *J* = 6.1 Hz, 2H, ArH), 2.52 (s, 3H, -CH_3_); ^13^C NMR (100 MHz, DMSO-*d^6^*) δ: 170.32, 150.92, 149.15, 148.75, 148.53, 139.37, 134.60, 134.56, 132.85, 131.15 130.21, 130.00, 129.04, 123.54, 123.40, 122.87, 120.84, 120.70, 56.49; IR (KBr) ν: 3066, 2917, 2757 (-N—H), 1279–1600 (-C=N), 1426 (-C=S) cm^−1^.

4-(2-methylacridin-9-yl)-5-phenyl-2,4-dihydro-3H-1,2,4-triazole-3-thione (**5g**): White powder, Yield 78%, m.p. 292–293 °C; ESI-MS m/z: 369 ([M + H]^+^); ^1^H NMR (400 MHz, DMSO-*d^6^*) δ: 14.60 (s, 1H, -NH), 8.26 (d, *J* = 8.8 Hz, 1H, ArH), 8.20 (d, *J* = 8.9 Hz, 1H, ArH), 7.90–7.82 (m, 1H, ArH), 7.77 (d, *J* = 9.0 Hz, 1H, ArH), 7.72–7.56 (m, 2H, ArH), 7.48 (s, 1H, ArH), 7.30–7.17 (m, 1H, ArH), 7.13 (d, *J* = 4.4 Hz, 4H, ArH), 2.51 (s, 3H, -CH_3_); ^13^C NMR (100 MHz, DMSO-*d^6^*) δ: 169.73, 151.47, 148.71, 148.47, 139.08, 135.20, 134.51, 131.29, 131.07, 130.11, 129.91, 129.28, 128.83, 127.33, 125.66, 123.71, 123.58, 123.02, 120.86, 22.15; IR (KBr) ν: 3066, 2917, 2757 (-N—H), 1279–1600 (-C=N), 1426 (-C=S) cm^−1^.

4-(4-methoxyphenyl)-4-(2-methylacridin-9-yl)-2,4-dihydro-3H-1,2,4-triazole-3-thione (**5h**): White powder, Yield 83%, m.p. 268–269 °C; ESI-MS m/z: 399 ([M + H]^+^); ^1^H NMR (400 MHz, DMSO-*d^6^*) δ: 14.48 (s, 1H, -NH), 8.27 (d, *J* = 8.8 Hz, 1H), 8.21 (d, *J* = 8.9 Hz, 1H, ArH), 7.86 (t, 1H, ArH), 7.78 (dd, *J* = 9.0, 1.5 Hz, 1H, ArH), 7.65 (d, *J* = 6.5 Hz, 1H, ArH), 7.60 (d, *J* = 8.5 Hz, 1H, ArH), 7.46 (s, 1H, ArH), 7.05 (d, *J* = 8.9 Hz, 2H, ArH), 6.68 (d, *J* = 8.9 Hz, 2H, ArH), 3.57 (s, 3H, -OCH_3_), 2.52 (s, 3H, -CH_3_); ^13^C NMR (100 MHz, DMSO-*d^6^*) δ: 169.51, 161.33, 151.35, 148.77, 148.53, 139.05, 135.42, 134.49, 131.06, 130.06, 128.85, 123.71 123.02, 120.86, 117.78, 114.78, 55.61, 22.17; IR (KBr) ν: 3095, 2925, 2750 (-N—H), 1360–1613 (-C=N), 1514 (-C=S) cm^−1^.

4-(2-methylacridin-9-yl)-5-(4-nitrophenyl)-2,4-dihydro-3H-1,2,4-triazole-3-thione (**5i**): Light yellow powder, Yield 53%, m.p. 245–247 °C; ESI-MS m/z: 414 ([M + H]^+^); ^1^H NMR (400 MHz, DMSO-*d^6^*) δ: 14.79 (s, 1H, -NH), 8.24 (t, *J* = 9.3 Hz, 2H, ArH), 8.02 (d, *J* = 8.9 Hz, 2H, ArH), 7.83 (s, 1H, ArH), 7.71–7.59 (m, 2H, ArH), 7.57 (d, *J* = 8.7 Hz, 1H, ArH), 7.44 (d, *J* = 8.9 Hz, 2H, ArH), 6.91 (s, H, ArH), 2.51 (s, 3H, -CH_3_), ^13^C NMR (100 MHz, DMSO-*d^6^*) δ: 169.99, 150.53, 148.55, 139.72, 135.26, 131.39, 130.41, 129.19, 128.69, 126.71, 124.62, 123.78, 123.49, 120.98, 22.19; IR (KBr) ν: 3054, 2918, 2755 (-N—H), 1432-1633 (-C=N), 1376 (-C=S) cm^−1^.

4-(4-chlorophenyl)-4-(2-methylacridin-9-yl)-2,4-dihydro-3H-1,2,4-triazole-3-thione (**5j**): Yellow powder, Yield 80%, m.p. 259–263 °C; ESI-MS m/z: 403 ([M + H]^+^); ^1^H NMR (400 MHz, DMSO-*d^6^*) δ: 14.65 (s, 1H, -NH), 8.27 (d, *J* = 8.8 Hz, 1H, ArH), 8.20 (d, *J* = 8.9 Hz, 1H, ArH), 7.88 (dd, *J* = 10.5, 4.0 Hz, 1H, ArH), 7.77 (d, *J* = 8.8 Hz, 1H, ArH), 7.73–7.57 (m, 2H, ArH), 7.49 (s, 1H, ArH), 7.24 (d, *J* = 8.5 Hz, 2H, ArH), 7.14 (d, *J* = 8.5 Hz, 2H, ArH); ^13^C NMR (100 MHz, DMSO-*d^6^*) δ: 169.85, 150.49, 148.74, 148.52, 139.19, 136.15, 134.86, 134.52, 131.08, 130.17, 129.97, 129.55, 129.00, 124.51, 123.56, 122.98, 120.81, 22.15; IR (KBr) ν: 3097, 2933 (-N—H), 1258-1600 (-C=N), 1497 (-C=S) cm^−1^.

#### 3.1.5. General Procedure for the Synthesis of Acridinyl 1,3,4-thiadiazol Derivatives (**6**)

About 3 mL of 98% concentrated sulfuric acid was added to a 50 mL round-bottom flask and stirred in an ice bath for 10 min at 0 °C. Then, intermediate **4** (0.5 mmol) was added into the solution in small portions over the course of 1 h. The reaction was continued at room temperature for 24–48 h, and 10 mL pure water was slowly added to reaction mixture in an ice bath. The final product **6** was precipitated, filtered off, washed with water, dried, and crystallized from ethyl alcohol.

7-methoxy-N-(5-(pyridin-4-yl)-1,3,4-thiadiazol-2-yl)-10,10a-dihydroacridin-9(8aH)-imine (**6a**): Orange solids, Yield 80%, m.p. 272–275 °C; ESI-MS m/z: 386 ([M + H]^+^); ^1^H NMR (400 MHz, DMSO-*d^6^*), δ: 8.78 (d, *J* = 5.7 Hz, 2H, ArH), 8.22 (d, *J* = 8.6 Hz, 1H, ArH), 8.00–7.94 (m, 3H, ArH), 7.90 (d, *J* = 6.2 Hz, 2H, ArH), 7.75 (dd, *J* = 9.3, 2.7 Hz, 1H, ArH), 7.58 (s, 1H, ArH), 7.53 (dt, *J* = 8.3, 4.0 Hz, 1H, ArH), 3.86 (s, 3H, -OCH_3_); ^13^C NMR (DMSO-*d^6^*, 100 MHz) δ: 156.40, 149.19, 140.03, 135.03, 130.65, 128.43, 127.65, 127.49, 127.29, 126.86, 125.02, 121.72, 119.38, 103.95, 56.20; IR (KBr) ν: 3055, 3011, 2837 (-C—H, -N—H), 1379-1631 (-C=N) cm^−1^.

7-methoxy-N-(5-phenyl-1,3,4-thiadiazol-2-yl)-10,10a-dihydroacridin-9(8aH)-imine (**6b**): Orange- yellow solid, Yield 81%, m.p. 263–265 °C; ESI-MS m/z: 385 ([M + H]^+^); ^1^H NMR (400 MHz, DMSO-*d^6^*), δ: 8.19 (d, *J* = 8.7 Hz, 1H, ArH), 7.92 (q, *J* = 9.3 Hz, 3H, ArH), 7.80 (dd, *J* = 6.6, 2.9 Hz, 2H, ArH), 7.68 (dd, *J* = 9.2, 2.7 Hz, 1H, ArH), 7.58 (s, 1H, ArH), 7.54–7.44 (m, 4H, ArH), 3.83 (s, 3H, -OCH_3_); ^13^C NMR (101 MHz, DMSO-*d^6^*) δ: 156.06, 140.22, 136.58, 134.49, 131.24, 130.46, 129.83, 127.72, 126.95, 126.79, 124.37, 121.59, 120.02, 120.33, 119.26, 116.76, 104.39, 56.03; IR (KBr) ν: 2771 (-C—H, N—H), 1329-1632 (-C=N) cm^−1^.

7-methoxy-N-(5-(4-methoxyphenyl)-1,3,4-thiadiazol-2-yl)-10,10a-dihydroacridin-9(8aH)-imine (**6c**): Orange-red solid, Yield 54%; m.p. 230–232 °C; ESI-MS m/z: 415 ([M + H]^+^); ^1^HNMR (400 MHz, DMSO-*d^6^*) δ: 8.35 (d, *J* = 8.5 Hz, 1H, ArH), 8.10 (d, *J* = 8.1 Hz, 3H, ArH), 8.01 (d, *J* = 9.8 Hz, 1H, ArH), 7.96 (s, 1H, ArH), 7.91–7.79 (m, 1H, ArH), 7.76–7.55 (m, 3H, ArH), 7.06 (t, *J* = 9.1 Hz, 1H, ArH), 3.93 (s, 3H, -OCH_3_), 3.81 (s, 3H, -OCH_3_); ^13^C NMR (101 MHz, DMSO-*d^6^*) δ: 156.91, 149.55, 144.54, 140.78, 136.88, 135.34, 129.01, 128.50, 126.77, 124.39, 121.00, 116.85, 112.99, 103.39, 103.21, 56.65, 56.17; IR (KBr) ν: 2771 (-C—H, -N—H), 1567 (-C=N) cm^−1^.

7-methoxy-N-(5-(4-nitrophenyl)-1,3,4-thiadiazol-2-yl)-10,10a-dihydroacridin-9(8aH)-imine (**6d**): Orange-red solid, Yield 33%; m.p. 258–260 °C; ESI-MS m/z: 430 ([M + H]^+^); ^1^H NMR (DMSO-*d^6^_,_* 400 MHz) δ: 8.32 (d, *J* = 8.9 Hz, 2H, ArH), 8.29 (d, *J* = 8.6 Hz, 1H, ArH), 8.04 (dd, *J* = 6.9, 4.8 Hz, 5H, ArH), 7.81 (dd, *J* = 9.3, 2.7 Hz, 1H, ArH), 7.61 (dd, *J* = 8.5, 2.5 Hz, 2H, ArH), 3.89 (s, 3H, -OCH_3_); ^13^C NMR (101 MHz, DMSO-*d^6^*) δ: 156.85, 148.76, 145.74, 142.43, 139.80, 136.85, 135.92, 135.35, 129.32, 127.93, 126.55, 125.67, 125.01, 121.90, 120.38, 119.60, 117.03, 103.59, 56.32; IR (KBr) ν: 2829 (-C—H, -N—H), 1346-1633 (-C=N) cm^−1^.

7-methoxy-N-(5-(4-chlorophenyl)-1,3,4-thiadiazol-2-yl)-10,10a-dihydroacridin-9(8aH)-imine (**6e**): Orange-yellow solid, Yield 51%; m.p. 203–205 °C; ESI-MS m/z: 419 ([M + H]^+^); ^1^H NMR (400 MHz, DMSO-*d^6^*) δ: 8.21 (d, *J* = 8.6 Hz, 1H, ArH), 7.94 (d, *J* = 8.1 Hz, 3H, ArH), 7.82 (s, 2H, ArH), 7.72 (dd, *J* = 9.2, 2.6 Hz, 1H, ArH), 7.58 (dd, *J* = 5.9, 2.6 Hz, 3H, ArH), 7.55–7.41 (m, 1H, ArH), 3.85 (s, 3H, -OCH_3_); ^13^C NMR (101 MHz, DMSO-*d^6^*) δ: 156.36, 140.09, 135.90, 134.8, 129.89, 129.19, 128.58, 128.25, 126.65, 124.87, 121.70, 120.01, 119.45, 116.90, 104.12, 102.60, 56.21; IR (KBr) ν: 2781 (-C—H, -N—H), 1467 (-C=N) cm^−1^.

7-methyl-N-(5-(pyridin-4-yl)-1,3,4-thiadiazol-2-yl)-10,10a-dihydroacridin-9(8aH)-imine **(6f**): Orange-yellow solid, Yield 43%; m.p. 276–278 °C; ESI-MS m/z: 370 ([M + H]^+^); ^1^H NMR (400 MHz, DMSO-*d^6^*) δ: 8.34 (d, *J* = 8.7 Hz, 2H, ArH), 8.23 (d, *J* = 8.9 Hz, 1H, ArH), 8.14 (d, *J* = 8.3 Hz, 1H, ArH), 8.05 (t, *J* = 9.3 Hz, 3H, ArH), 8.01–7.91 (m, 2H, ArH), 7.88 (s, 1H, ArH), 7.51 (d, *J* = 8.2 Hz, 1H, ArH), 2.48 (s, 3H, -CH_3_); ^13^C NMR (101 MHz, DMSO-*d^6^*) δ: 158.09, 155.78, 151.07, 140.54, 135.49, 133.66, 131.53, 127.77, 126.46, 123.78, 121.88, 118.38, 118.11, 116.14, 21.31; IR (KBr) ν: 2824 (-C—H, -N—H), 1347-1600 (-C=N) cm^−1^.

7-methyl-N-(5-phenyl-1,3,4-thiadiazol-2-yl)-10,10a-dihydroacridin-9(8aH)-imine (**6g**): Yellow solid, Yield 56%; m.p. 294–296 °C; ESI-MS m/z: 369 ([M + H]^+^); ^1^H NMR (400 MHz, DMSO-*d^6^*) δ: 8.17 (d, *J* = 8.5 Hz, 1H, ArH), 8.07 (s, 1H, ArH), 7.97–7.85 (m, 2H, ArH), 7.80 (d, *J* = 6.9 Hz, 4H, ArH), 7.59-7.49 (m, 3H, ArH), 7.48–7.34 (m, 1H, ArH), 2.45 (s, 3H, -CH_3_); ^13^C NMR (101 MHz, DMSO-*d^6^*) δ: 160.12, 157.49, 140.60, 138.98, 137.32, 135.07, 133.97, 131.26, 130.45, 129.82, 127.17, 127.15, 125.56, 124.06, 119.50, 119.21, 118.10, 117.00, 21.44; IR (KBr) ν: 2792 (C—H, N—H), 1366-1627 (C=N) cm^−1^.

7-methyl-N-(5-(4-methoxyphenyl)-1,3,4-thiadiazol-2-yl)-10,10a-dihydroacridin-9(8aH)-imine (**6h**): Orange-yellow solid, Yield 84%; m.p. >300 °C; ESI-MS m/z: 399 ([M + H]^+^); ^1^H NMR (400 MHz, DMSO-*d^6^*) δ: 8.33 (d, *J* = 8.7 Hz, 1H, ArH), 8.18 (s, 1H, ArH), 8.10–8.01 (m, 3H, ArH), 7.98 (d, *J* = 8.8 Hz, 2H, ArH), 7.91–7.77 (m, 1H, ArH), 7.68 (dd, *J* = 8.6, 2.3 Hz, 1H, ArH), 7.62 (dd, *J* = 14.4, 7.9 Hz, 1H, ArH), 7.08 (d, *J* = 8.7 Hz, 1H, ArH), 2.48 (s, 3H, -CH_3_); ^13^C NMR (101 MHz, DMSO-*d^6^*) δ: 162.17, 159.02, 140.47, 139.22, 137.04, 136.13, 135.63, 128.91, 127.11, 127.06, 126.92, 126.85, 125.57, 125.06, 120.71, 120.03, 119.59, 118.29, 117.35, 113.02, 56.20, 21.52; IR (KBr) ν: 3090, 2920, 2830 (-C—H, -N—H), 1486-1628 (-C=N) cm^−11^.

7-methyl-N-(5-(4-nitrophenyl)-1,3,4-thiadiazol-2-yl)-10,10a-dihydroacridin-9(8aH)-imine (**6i**): Purple-red solid, Yield 53%; m.p. 224–226 °C; ESI-MS m/z: 414 ([M + H]^+^); ^1^H NMR (400 MHz, DMSO-*d^6^*) δ: 12.17 (s, 1H, -NH), 8.35 (d, *J* = 8.9 Hz, 2H, ArH), 8.15 (d, *J* = 8.9 Hz, 2H, ArH), 7.96 (d, *J* = 5.1 Hz, 2H, ArH), 7.72 (t, *J* = 7.0 Hz, 1H, ArH), 7.65–7.56 (m, 2H, ArH), 7.54 (d, *J* = 8.5 Hz, 1H, ArH), 7.18 (t, *J* = 7.6 Hz, 1H, ArH), 2.35 (s, 3H, -CH_3_); ^13^C NMR (100 MHz, DMSO-*d^6^*) δ: 165.41, 148.81, 143.72, 140.52, 139.40, 138.81, 136.22, 135.69, 135.63, 127.93, 126.99, 125.65, 125.02, 120.12, 119.72, 118.15, 117.34, 113.14, 21.54; IR (KBr) ν: 2918 (-C—H, -N—H), 1340-1627 (-C=N) cm^−1^.

7-methyl-N-(5-(4-chlorophenyl)-1,3,4-thiadiazol-2-yl)-10,10a-dihydroacridin-9(8aH)-imine (**6j**): Orange-yellow solid, Yield 67%, m.p. 258–260 °C; ESI-MS m/z: 403 ([M + H]^+^); ^1^H NMR (400 MHz, DMSO-*d^6^*) δ: 8.23 (d, *J* = 8.6 Hz, 1H, ArH), 8.12 (s, 1H, ArH), 8.04–7.93 (m, 2H, ArH), 7.88 (s, 2H, ArH), 7.81 (d, *J* = 8.5 Hz, 2H, ArH), 7.58 (d, *J* = 8.5 Hz, 2H, ArH), 7.51 (t, *J* = 7.5 Hz, 1H, ArH), 2.49 (s, 3H, -CH_3_); ^13^C NMR (101 MHz, DMSO-*d^6^*) δ: 160.71, 146.36, 140.56, 139.09, 137.87, 135.87, 135.50, 129.89, 129.14, 128.58, 127.04, 125.34, 124.71, 119.72, 119.37, 118.16, 117.13, 21.47; IR (KBr) ν: 2795 (-C—H, -N—H), 1366–1627 (-C=N) cm^−1^.

#### 3.1.6. Preparation of Single Crystal Compounds **5b** and **6d** and Their X-ray Single Crystal Diffraction Method

A single crystal of **5b** and **6d** suitable for X-ray diffraction study was cultivated from 95% ethyl alcohol and N, N-dimethylformamide respectively, by a slow evaporation method at room temperature. All measurements were performed with Mo Kα radiation (λ = 0.7107 Å) on a Brucker SMART 1000 CCD X diffractometer (Billerica, MA, USA). The structure was solved by direct methods with SHELXS-97 [25] and refined by SHELXL-97 [26]. All non-hydrogen atoms were refined with anisotropic thermal parameters. The final full-matrix least-squares refinement of **5b** gave R = 0.0914, ω = 1/[s^2^(Fo^2^) + (0.0431 *p*)^2^ + 0.2721 *p*] where *p* = (Fo^2^ + 2Fc^2^)/3, S = 1.043, (Δ/*σ*)_max_ = 0.237 and (Δ/σ)_min_ = −0.267 e/Å^3^. In addition, the final full-matrix least-squares refinement of **6d** gave R = 0.0914, ω = (1/[s^2^(Fo^2^) + (0.0650 *p*)^2^ + 0.0224 *p*] where *p* = (Fo^2^ + 2Fc^2^)/3, S = 1.028, (Δ/*σ*)_max_ = 0.236 and (Δ/*σ*)_min_ = −0.197 e/Å^3^.

### 3.2. In-Vivo Antitumor Activity

#### 3.2.1. Antiproliferative Activity

Test samples, including compounds **4**–**6** and commercial classical anticancer drugs (5-FU and cis-platinum), were screened for their anti-cancer activity against HFF human foreskin fibroblast cells, MGC-803 human gastric cancer cells, BEL-7404 human hepatocellular carcinoma cells, NCI-H460 human large cell carcinoma cells, and T24 human bladder carcinoma cells using the 3-(4,5-dimethylthiazol-2-yl)-2,5-diphenyltetrazoliumbromide (MTT) assay method cited in the literature [27]. The initial concentration of all the test samples was 100 μg/mL, which was serially diluted in complete medium with ten-fold dilutions to give six concentrations per compound. Their cytotoxicity was determined in 96-well flat bottomed microtiter plates. All the test samples were tested in triplicate. The results were expressed as the drug concentration that inhibited cell growth by 50% as compared to the controls (IC_50_). The IC_50_ values were calculated from regression lines obtained from the percent cell growth inhibition plotted as a function of the logarithm of the dose.

#### 3.2.2. Apoptosis and Cell Cycle Analysis

The apoptosis assay and the cell cycle analysis were carried out by cytometry (FACSVerse, BD, Piscataway, NJ, USA) at an excitation wavelength of 488 nm according to the method described in the literature with slight modifications [28]. The cells were seeded at 2 × 10^6^/well and washed by cold PBS. The buffer solutions were prepared using 0.1 M pH 7.4 Hepes/NaOH, 1.4 M NaCl, and 25 mM CaCl_2_.

#### 3.2.3. Topo I Inhibitory Activity

Topo I and pBR322 were obtained commercially from Takara Bio Inc. (Shiga, Japan). And the enzyme inhibitory activity was determined by our previous methods [19].

#### 3.2.4. Anti-Angiogenic Effect Using the Zebrafish Model

(a) Zebrafish toxicity assay

The zebrafish embryos were collected at 6 hpf and randomly divided into naive control (embryos maintained in distilled water), vehicle control (embryos treated with 2% DMSO), and drug groups. Stock solutions of all drugs were prepared in 2% DMSO as a solubilizing agent and diluted to three concentrations (0.5 mg/mL, 1 mg/mL, and 2 mg/mL). Each group had 20 embryos per test concentration. The zebrafish embryos were maintained in an incubator at 28 °C and read at 72 hpf for their mortality and teratogenicity (including non-hatching, egg condensation, spinal curvature, pericardial enlargement, etc.). Each compound was evaluated in three independent biological experiments.

(b) Angiogenesis assay

The 24 hpf zebrafish embryos were dechorionated with a 1 gL^−1^ pronase treatment and maintained in distilled water in 12-well cell culture plates (each well contained 20 embryos). A negative control group containing zebrafish embryos in distilled water and a vehicle treatment group that was treated with 2% dimethyl sulfoxide (DMSO) were prepared. The anti-angiogenic compounds were diluted to 1 mg/mL. After incubating at 28 °C for 72 h, the embryos were immersed in 4% paraformaldehyde and dehydrated by gradient ethanol. Subsequently, the embryos were balanced in NTMT buffer (5 M NaCl + 1 M Tris [pH 9.0–9.5] + 1 M MgCl_2_+ 10% Tween), and nitrotetrazolium blue chloride (NBT) and p-toluidine salt (BCIP) staining were performed. To evaluate the effect of compounds on the angiogenesis of zebrafish embryos, the growth of embryonic sub-intestinal veins (SIVs) at 72 hpf was observed using an IX71 Olympus microscope (Hamburger, Germany). The length of the SIVs was calculated using the image J 1.8.0 software (Bethesda, MD, USA).

## 4. Conclusions

A new series of acridine-triazole and acridine-thiadiazole derivatives were synthesized and characterized by spectral studies. All the synthesized compounds were evaluated for their in vitro cytotoxic activities against HFF, MGC-803, BEL-7404, NCI-H460, and T24 by the MTT assay method. Most of the compounds were sensitive to MGC-803 and T24 cell lines. Compared to all the prepared compounds, **4a**, **5d** and **6h** exhibited the best anticancer activity against MGC-803 cell lines, and compounds **4h**, **5h** and **6h** showed the most excellent antitumor activity against T24. Preliminary studies of antitumor mechanisms revealed that the representative compounds (**5d** and **6h** or **4h** and **6h**) could suppress cell proliferation by inducing apoptosis in the Q3 period of MGC-803 or T24 cell lines. Compound **5d** might inhibit the growth of tumor cells by arresting cells in the G2 phase, while compound **4h** had a great effect on the S phase. In the zebrafish experiment, compound **5d** displayed a superior antiangiogenic effect and lower toxicity than other compounds. Therefore, compound **5d** has the potential to be an antitumor drug with high efficiency and low toxicity.

## Figures and Tables

**Table 1:** Effect of compounds **4**, **5** and **6** against cell viability of different cell lines ^#^ (μM).

No.	HFF	MGC-803	BEL-7404	NCI-H460	T24	LO2
**4a**	75.79 ± 3.52	10.89 ± 1.82	26.93 ± 2.58	36.41 ± 3.12	>100	21.96 ± 1.71
**4b**	72.56 ± 3.57	14.47 ± 2.06	25.78 ± 2.83	42.27 ± 2.15	29.82 ± 2.85	34.37 ± 2.05
**4c**	63.74 ± 2.59	21.04 ± 1.55	13.33 ± 1.37	52.37 ± 3.67	11.23 ± 2.51	19.45 ± 1.69
**4d**	65.28 ± 3.82	34.99 ± 3.57	43.91 ± 2.59	40.54 ± 4.52	13.01 ± 1.64	25.12 ± 1.33
**4e**	66.84 ± 4.52	25.55 ± 1.97	25.95 ± 2.17	73.25 ± 3.67	10.32 ± 1.07	10.23 ± 1.12
**4f**	55.21 ± 1.36	11.24 ± 0.96	34.37 ± 2.24	53.66 ± 3.04	9.66 ± 1.54	10.08 ± 0.96
**4g**	70.11 ± 3.97	13.54 ± 1.59	20.17 ± 4.13	39.01 ± 2.05	25.84 ± 1.51	14.34 ± 1.52
**4h**	>100	11.25 ± 1.46	27.10 ± 2.91	25.36 ± 3.16	8.05 ± 1.06	9.01 ± 0.93
**4i**	60.73 ± 2.31	22.34 ± 1.35	23.32 ± 1.33	60.40 ± 2.95	9.89 ± 1.45	11.76 ± 1.38
**4j**	61.53 ± 1.85	16.37 ± 1.56	24.45 ± 3.53	32.22 ± 2.36	19.95 ± 1.32	20.53 ± 1.39
**5a**	74.50 ± 4.03	22.41 ± 1.32	22.06 ± 2.72	36.45 ± 2.96	21.17 ± 2.72	41.99 ± 2.31
**5b**	53.58 ± 2.78	15.81 ± 1.94	27.65 ± 2.97	42.08 ± 3.74	22.05 ± 1.85	58.28 ± 3.25
**5c**	75.51 ± 2.92	15.13 ± 0.98	28.71 ± 2.24	36.45 ± 3.92	29.29 ± 1.91	>100
**5d**	62.93 ± 1.90	5.52 ± 1.04	25.07 ± 2.89	19.44 ± 1.58	15.92 ± 1.38	51.79 ± 3.46
**5e**	74.93 ± 3.35	8.50 ± 1.85	34.66 ± 2.64	35.13 ± 1.94	18.45 ± 1.64	>100
**5f**	69.22 ± 2.16	15.24 ± 1.08	44.21 ± 2.68	58.79 ± 3.22	15.72 ± 1.58	46.78 ± 2.93
**5g**	68.31 ± 2.74	19.35 ± 1.38	20.54 ± 1.13	30.64 ± 2.21	19.36 ± 2.17	44.55 ± 2.35
**5h**	68.07 ± 2.64	10.88 ± 0.97	40.33 ± 2.06	26.32 ± 2.51	11.25 ± 1.16	37.67 ± 2.47
**5i**	70.85 ± 2.99	8.92 ± 0.99	31.66 ± 2.36	28.31 ± 1.32	14.26 ± 1.27	>100
**5j**	64.69 ± 3.36	13.51 ± 1.91	45.87 ± 2.48	21.78 ± 2.46	13.06 ± 1.70	36.44 ± 2.65
**6a**	68.82 ± 1.87	14.31 ± 1.29	19.21 ± 1.30	25.34 ± 3.57	10.18 ± 0.96	40.24 ± 2.74
**6b**	79.32 ± 2.48	23.27 ± 1.97	32.29 ± 2.82	47.51 ± 3.51	49.36 ± 4.59	>100
**6c**	65.21 ± 3.92	12.13 ± 1.22	25.11 ± 2.15	30.23 ± 2.45	24.27 ± 2.34	>100
**6d**	>100	26.66 ± 3.35	>100	>100	>100	>100
**6e**	44.71 ± 1.44	9.01 ± 1.32	21.33 ± 2.81	27.88 ± 3.97	14.88 ± 1.30	33.64 ± 2.01
**6f**	45.38 ± 2.18	12.35 ± 1.96	40.26 ± 2.19	55.72 ± 3.28	13.86 ± 1.37	37.22 ± 2.12
**6g**	76.45 ± 2.79	9.95 ± 1.03	31.25 ± 3.27	25.87 ± 1.83	19.33 ± 1.05	>100
**6h**	33.90 ± 1.28	6.85 ± 0.84	20.25 ± 1.59	13.33 ± 1.39	8.93 ± 1.25	43.77 ± 2.63
**6i**	91.95 ± 2.99	22.92 ± 1.85	43.66 ± 2.36	36.41 ± 3.15	29.89 ± 2.45	>100
**6j**	56.23 ± 3.16	12.99 ± 1.89	48.47 ± 3.06	13.88 ± 1.83	15.47 ± 1.98	51.17 ± 3.09
**5-FU**	25.45 ± 1.27	30.45 ± 2.87	34.52 ± 1.18	44.04 ± 0.54	32.04 ± 1.23	40.15 ± 1.65
**cis-platinum**	10.85 ± 0.34	15.97 ± 1.53	10.01 ± 0.52	7.126 ± 1.24	9.13 ± 1.54	21.38 ± 1.25

^#^ human foreskin fibroblasts (HFF); human gastric cancer cells-803 (MGC-803); hepatocellular carcinoma bel-7404 (BEL-7404); large cell lung cancer cells (NCI-H460); and bladder cancer cells (T24); LO2 human normal liver cells (LO2).

**Table 2:** The mortality rate (MOR) and malformation rate (MAR) of some of the selected compounds.

NO.	Sample Concentration									
	Control		2% DMSO		2 mg/mL		1.5 mg/mL		1 mg/mL	
	MOR	MAR	MOR	MAR	MOR	MAR	MOR	MAR	MOR	MAR
**4a**	0	0	0	0	45% ^c^	55% ^c^	40% ^c^	60% ^c^	15% ^c^	85% ^c^
**4b**	0	0	0	0	40% ^a^	55% ^a^	15% ^a^	70% ^a^	15% ^a^	70% ^a^
**4c**	0	0	0	0	35% ^a^	50% ^a^	25% ^a^	60% ^a^	45% ^a^	50% ^a^
**4f**	0	0	0	0	85% ^b^	15% ^b^	20% ^b^	80% ^b^	10% ^b^	60% ^b^
**4h**	0	0	0	0	65% ^a^	35% ^a^	15% ^a^	85% ^a^	15% ^a^	35% ^a^
**5d**	0	0	0	0	0	10% ^c^	0	10% ^a^	0	5% ^b^
**5h**	0	0	0	0	0	15% ^a^	0	10% ^a^	0	5% ^a^
**5j**	0	0	0	0	0	15% ^a^	0	5% ^a^	0	0
**6a**	0	0	0	0	5% ^b^	90% ^b^	0	95% ^b^	0	15% ^b^
**6e**	0	0	0	0	65% ^c^	35% ^c^	30% ^c^	70% ^c^	10% ^c^	10% ^c^
**6h**	0	0	0	0	15% ^b^	65% ^b^	0	80% ^b^	0	35% ^b^
**6i**	0	0	0	0	0	25% ^c^	0	10% ^b^	0	0

Note: ^a^ = *p* < 0.01; ^b^ = *p* < 0.001; ^c^ = *p* < 0.002, compared with the control group.

## Data Availability

We have presented all of our main data in the form of tables and figures. CCDC 2214949 contain supplementary crystallographic data for compound **5b** and CCDC 2214923 contain supplementary crystallographic data for compound **6d**. These datas can be obtained free of charge via http://www.ccdc.cam.ac.uk/conts/retrieving.html (accessed on 20 December 2022) or the Cambridge Crystallographic Data Centre, 12 Union Road, Cambridge CB2 1EZ, UK; Fax: +44-1223-336-033; or e-mail: deposit@ccdc.cam.ac.uk.
